# *Acetobacter indonesiensis* Pneumonia after Lung Transplantation

**DOI:** 10.3201/eid2403.170409

**Published:** 2018-03

**Authors:** Sankha S. Basu, Mary L. Delaney, Ning Li, Andrew Bruce Onderdonk, Lynn Bry

**Affiliations:** Brigham and Women’s Hospital, Boston, Massachusetts, USA

**Keywords:** *Acetobacter* *indonesiensis*, pneumonia, lung transplant, 16S rRNA, acetic acid, fever, leukocytosis, anemia, thrombocytopenia, acute kidney damage, respiratory infections, bacteria, antimicrobial resistance

## Abstract

We report a case of *Acetobacter* *indonesiensis* pneumonia in a 51-year-old woman after bilateral lung transplantation. We found 2 other *A*. *indonesiensis* pneumonia cases reported in the literature. All 3 cases involved complex patients exposed to broad-spectrum antimicrobial drugs, suggesting that this pathogen may be opportunistic and highly drug-resistant.

A 51-year-old woman who had a medical history of hypersensitivity pneumonitis, extrinsic allergic alveolitis, and short telomere syndrome was admitted to a local hospital in Massachusetts, USA, for hypoxemic respiratory failure. The patient was transferred to the tertiary care hospital in which we practice in Boston, where she ultimately underwent a bilateral lung transplant from a high-risk donor without induction immunosuppression. The donor lungs grew group C *Streptococcus*, *Peptostreptococcus micros*, and *Candida albicans*. The native lungs were culture-negative. 

The patient’s postoperative hospital course was complicated by fever, leukocytosis, anemia, thrombocytopenia, and acute kidney injury. The clinical treatment team treated the patient with trimethoprim/sulfamethoxazole (TMP/SMX) and vancomycin; the latter was discontinued and piperacillin/tazobactam (14 days total) was administered after identification of *P. micros* in the donor’s lungs. On postoperative day 21, 4 days after completion of her antimicrobial drug therapy, the patient continued to have respiratory symptoms, and we cultured samples from a tracheostomy suction. A Gram stain of the tracheostomy suction fluid revealed gram-variable coccobacilli. The next day, we also found 2 bronchioalveolar lavage specimens to be positive for gram-variable coccobacilli and considered them to be of the same phenotype. The patient’s symptoms, along with the presence of the organism in 3 separate and sequential samples, argued against contamination.

Standard microbiological culture techniques revealed a slow-growing organism that was catalase-positive, oxidase-negative, L-pyrrolidonyl-β-napthylamide hydrolysis–negative, and vancomycin-resistant. We did not identify the organism by using exhaustive phenotypic techniques. We sequenced 16S rRNA (Technical Appendix Table) and identified the organism as *Acetobacter indonesiensis*; we deposited this sequence in GenBank (accession no. KP330469). Because of the rare occurrence of this pathogen in humans (*1*,*2*), we achieved additional biochemical testing by using short- and medium-chain fatty acid analysis, which provided additional evidence supporting sequence-based identification. 

At the time of the infection, the clinical microbiology laboratory at our hospital was not equipped with a matrix-assisted laser desorption/ionization time-of-flightmatrix-assisted laser desorption/ionization time-of-flight matrix-assisted laser desorption/ionization time-of-flight matrix-assisted laser desorption/ionization time-of-flight mass spectrometry bacterial identification platform. However, this organism is not in any Food and Drug Administration–approved databases and therefore would not have been identified by using this instrumentation. Antimicrobial drug susceptibility testing using disk diffusion revealed an organism that failed to demonstrate in vitro activity to ampicillin, levofloxacin, ciprofloxacin, cephalothin, cefotetan, ceftadazime, cefepime, chloramphenicol, ertapenem, meropenem, piperacillin, aztreonam, thiosulfil/sulfamethizole, TMP/SMX, or colistin. The isolate did, however, demonstrate in vitro activity against aminoglycosides, tetracyclines, imipenem, and ceftriaxone. This drug susceptibility profile was similar to the profile found against the *A. indonesiensis* organism identified in a previously reported case (*2*). 

Although this patient’s isolate was resistant to the antimicrobial drugs she had received, her symptoms ultimately resolved. On postoperative day 33, her respiratory function had improved, and she was prescribed TMP/SMX (prophylaxis) and fluconazole at discharge. 

At the time the bacteria was speciated by 16S rRNA gene sequencing, the patient’s infection had already resolved. The clinical record does not document any additional antimicrobial treatments she may have received from other clinical teams, including the infectious disease, transplant, and nephrology departments. This organism appeared in 3 consecutive respiratory specimens collected when the patient’s symptoms worsened and raised concerns among the attending clinical teams of potential infection with an innately drug-resistant species. However, we cannot definitively rule out the potential for colonization because a combination of factors likely led to clinical improvement in the patient. The organism was not detected in any subsequent bronchoscopies.

The genus *Acetobacter* encompasses a group of acetic acid–producing organisms that can survive at low pH, largely occupy environmental niches, are used industrially to produce acetic acid products, and are not generally thought to be human pathogens (*1*). Analysis of the medical literature revealed 2 other documented clinical cases of *A. indonesiensis* infection among humans (*2*,*3*). The first case involved a patient with cystic fibrosis who had undergone a recent lung transplant (*2*). Similar to our case-patient, the patient had undergone bilateral lung transplants and *A. indonesiensis* pneumonia subsequently developed in both after a long course of broad-spectrum antimicrobial drugs. The second case involved a child with metachromatic leukodystrophy who was found to have *A. indonesiensis* bacteremia after extensive nursing care and invasive devices, including a port catheter thought to be the source of the infection (*3*). As with the patient we report, the patient in that report had been treated with a 2­-week course of pipericillin/tazobactam, although her initial diagnosis was bacteremia rather than pneumonia. 

The case of *A. indonesiensis* human infection we report and both previous cases we found in the literature involved chronically ill patients with complex medical conditions who were exposed to a long course of broad-spectrum antimicrobial drugs. Although the source of the infecting organism in all 3 cases could not be definitively determined, the similarities between the cases raise the possibility that *A. indonesiensis* may represent a novel and emerging opportunistic and highly drug-resistant pathogen. Furthermore, the use of specific genotypic techniques such as 16S rRNA sequencing may aid in the identification of environmental organisms that are not identified by using traditional microbiological techniques.

Technical Appendix Genetic and biochemical testing of the isolate, including 16S sequencing results, a phylogenetic tree showing the position of the isolate, short chain fatty acid analysis, and antibiotic susceptibility testing results.
