# Supplementary material for: Acetobacter indonesiensis Pneumonia after Lung Transplantation
Source: Emerg Infect Dis. 2018 Mar;24(3):598–9. doi: 10.3201/eid2403.170409 (PMC5823329; doi:10.3201/eid2403.170409)
Supplement: Technical Appendix — Genetic and biochemical testing of the isolate, including 16S sequencing results, a phylogenetic tree showing the position of the isolate, short chain fatty acid analysis, and antibiotic susceptibility testing results. [file 17-0409-Techapp-s1.pdf]

Article DOI: https://doi.org/10.3201/eid2403.170409

Acetobacter indonesiensis Pneumonia after Lung Transplantation

Technical Appendix

16S rRNA sequencing

For this analysis, the full-length 16S rRNA gene was amplified and sequenced in a set of 6 reactions to cover the entire length. All 6 sequencing reactions were successful, yielding a contig of 1,349 base pairs (Genbank accession no. KP330469). A search of the Isentio RipSeq 16S database yielded a very strong match to Acetobacter indonesiensis with 99.3–100% identity and 0.8% difference separating it from other species. CLSI guidelines specify identity cut-offs for members of the alpha-Proteobacteria of 0.8% separation between species, thus making it is possible to make an identity to the species level. To more accurately identify the organisms and its phylogenetic relationships with the other Acetobacter species, maximum likelihood tree of 16S rRNA was built by using FastTree (*1*). To build the phylogenetic tree (Figure 1), 16S rRNA full length sequences were collected from Ribosomal Database Project (*2*), including 9 Acetobacter species, and 2 other species as out groups and multiple sequence alignment was done by Muscle v3.8.31 (*3*).

Additional Biochemical Analysis

Additional biochemical analysis was pursued to confirm the identity of the organism, due to its rare occurrence in humans. Identification using long chain fatty acid analysis (Microbial Identification System (MIS), MIDI, Inc.) revealed a fatty acid profile matching most closely with Gluconacetobacter (Acetobacter) liquifaciens (0.483), Acetobacter pasteurianus (0.477) and Acetobacter aceti (0.368), species closely related to Acetobacter indonesisensis (which is not in the MIS library database). The organism was found to grow best at 30°C in tryptic soy broth. Notably, short chain fatty acid analysis of the cultured media at 48 h revealed a large chromatographic peak corresponding to acetate when the growth media was supplemented with 5% ethanol (Technical Appendix Figure 2). Antibiotic susceptibility testing was performed on

sheep's blood agar plates, as the organism failed to grow on Mueller Hinton plates (Techncial Appendix Table).

References

1. Yamada Y, Hosono R, Lisdyanti P, Widyastuti Y, Saono S, Uchimura T, et al. Identification of acetic acid bacteria isolated from Indonesian sources, especially of isolates classified in the genus Gluconobacter. J Gen Appl Microbiol. 1999;45:23–8. http://dx.doi.org/10.2323/jgam.45.23

2. Bittar F, Reynaud-Gaubert M, Thomas P, Boniface S, Raoult D, Rolain JM. Acetobacter indonesiensis pneumonia after lung transplant. Emerg Infect Dis. 2008;14:997–8. http://dx.doi.org/10.3201/eid1406.071236

3. Kohlmann R, Barenberg K, Anders A, Gatermann SG. Acetobacter indonesiensis bacteremia in child with metachromatic leukodystrophy. Emerg Infect Dis. 2016;22:1681–3. http://dx.doi.org/10.3201/eid2209.160566

4. Price MN, Dehal PS, Arkin AP. FastTree 2—approximately maximum-likelihood trees for large alignments. PLoS One. 2010;5:e9490. http://dx.doi.org/10.1371/journal.pone.0009490

5. Cole JR, Wang Q, Fish JA, Chai B, McGarrell DM, Sun Y, et al. Ribosomal Database Project: data and tools for high throughput rRNA analysis. Nucleic Acids Res. 2014;42(D1):D633–42. http://dx.doi.org/10.1093/nar/gkt1244

6. Edgar RC. MUSCLE: multiple sequence alignment with high accuracy and high throughput. Nucleic Acids Res. 2004;32:1792–7. http://dx.doi.org/10.1093/nar/gkh340

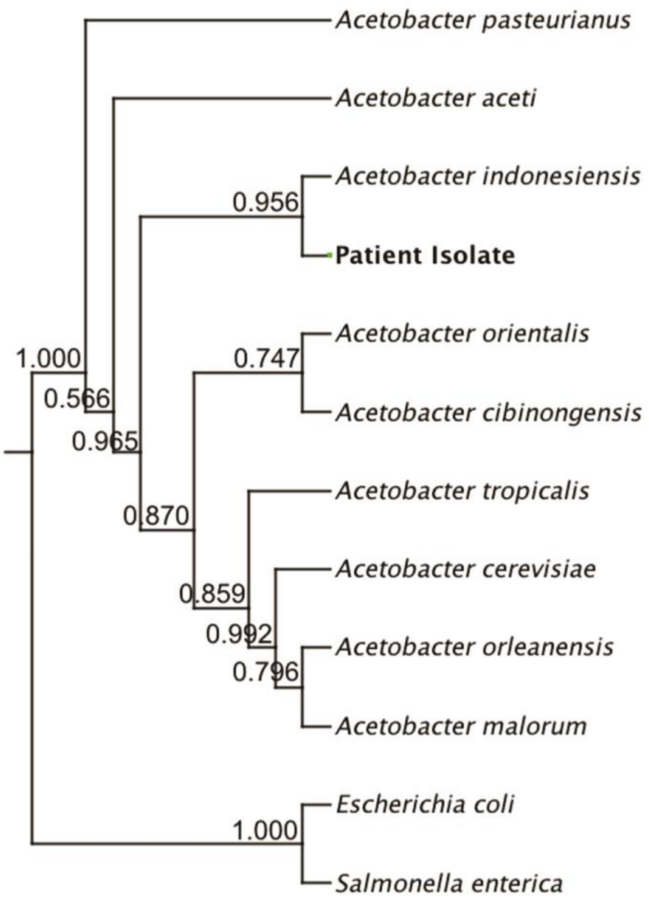

Technical Appendix Figure 1. Phylogenetic tree showing the position of the patient isolate. The numbers on the branches are local support values to estimate the reliability of each split in the tree.

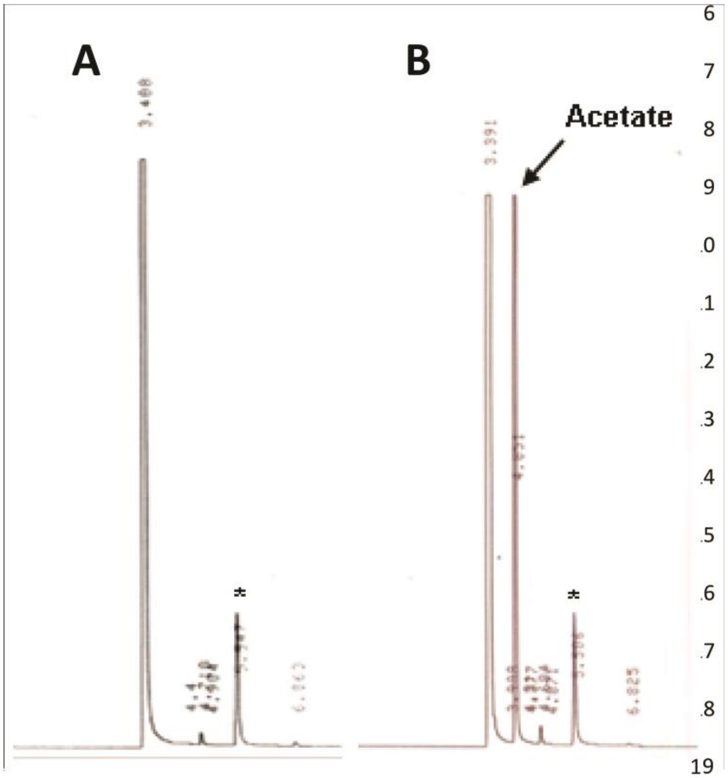

Technical Appendix Figure 2. Short chain fatty acid analysis using gas chromatography of Acetobacter indonesiensis grown at 30°C for 48 hours in tryptic soy broth containing A) no ethanol and B) 5% ethanol. Acetate peak labeled on chromatogram.*Internal standard peak.

Technical Appendix Table. Antibiotic susceptibility testing results (KB zone diameters).

Antimicrobial drug	mm
Amikacin	12
Amoxicillin/clavulanic acid	10
Ampicillin	6
Aztreonam	6
Cefepime	6
Cefotetan	6
Ceftadazime	6
Ceftriaxone	18
Cephalothin	6
Chloramphenicol	6
Ciprofloxacin	6
Colistin	6
Ertapenem	6
Gentamicin	10
Imipenem	14
Levofloxacin	6
Meropenem	6
Minocycline	30
Nitrofurantoin	6
Piperacillin	7
Tetracycline	34
Thiosulfil/Sulfamethizole	6
TMP/SMX	6
Tobramycin	10
